# Microencapsulation of a *Pseudomonas* Strain (VUPF506) in Alginate–Whey Protein–Carbon Nanotubes and Next-Generation Sequencing Identification of This Strain

**DOI:** 10.3390/polym13234269

**Published:** 2021-12-06

**Authors:** Fariba Fathi, Roohallah Saberi Riseh, Pejman Khodaygan, Samin Hosseini, Yury A. Skorik

**Affiliations:** 1 Department of Plant Protection, Faculty of Agriculture, Vali-e-Asr University of Rafsanjan, Imam Khomeini Square, Rafsanjan 7718897111, Iran; fariba.fathi@stu.vru.ac.ir (F.F.); r.saberi@vru.ac.ir (R.S.R.); Pkhodaygan@vru.ac.ir (P.K.); s.hosseini@vru.ac.ir (S.H.); 2 Institute of Macromolecular Compounds of the Russian Academy of Sciences, Bolshoi VO 31, 199004 St. Petersburg, Russia

**Keywords:** extrusion, emulsification, spray drying, alginate, whey protein, carbon nanotubes, *Pseudomonas*, whole genome sequence

## Abstract

Alginate is a common agent used for microencapsulation; however, the formed capsule is easily damaged. Therefore, alginate requires blending with other biopolymers to reduce capsule vulnerability. Whey protein is one polymer that can be incorporated with alginate to improve microcapsule structure. In this study, three different encapsulation methods (extrusion, emulsification, and spray drying) were tested for their ability to stabilize microencapsulated *Pseudomonas* strain VUPF506. Extrusion and emulsification methods enhanced encapsulation efficiency by up to 80% and gave the best release patterns over two months. A greenhouse experiment using potato plants treated with alginate–whey protein microcapsules showed a decrease in *Rhizoctonia* disease intensity of up to 70%. This is because whey protein is rich in amino acids and can serve as a resistance induction agent for the plant. In this study, the use of CNT in the ALG–WP system increased the rooting and proliferation and reduced physiological complication. The results of this study showed that the technique used in encapsulation could have a significant effect on the efficiency and persistence of probiotic bacteria. Whole genome sequence analysis of strain VUPF506 identified it as *Pseudomonas chlororaphis* and revealed some genes that control pathogens.

## 1. Introduction

Microencapsulation within a bio-polymeric matrix is a promising strategy for protecting probiotic bacteria against adverse conditions. In crop agriculture, the principle underlying probiotic bacterial microencapsulation is to preserve beneficial bacteria introduced into the soil and to ensure a controlled and prolonged release of those bacteria [1]. Probiotics, such as pathogen-antagonist bacteria, are a crucial part of agricultural soils, as these beneficial bacteria increase plant growth and control plant pathogens by several mechanisms. However, these bacteria have low stability in soil when exposed to adverse environmental conditions. Application of these agents is often inefficient; therefore, the use of an appropriate carrier could be effective in increasing the survival and stability of soil probiotic bacteria [2].

Various natural polymers have been used for the encapsulation of probiotic bacteria. The most commonly used bio-polymer for this purpose is alginate (ALG), which has recognized advantages that include non-toxicity, simplicity, biocompatibility, and low cost. However, ALG microcapsules have a crackled and porous structure that lowers their mechanical and chemical stability [3]. Blending ALG with other biopolymers is viewed as an optimal approach for strengthening the microcapsule structure and improving its physicochemical properties [4].

Whey protein (WP), which is a mixture of proteins isolated from whey as a by-product of cheese production, is a natural polymer used for the encapsulation of probiotics and is one of the candidates that can be combined with ALG. This combination therefore represents a promising approach for probiotic encapsulation in plant agriculture [5]. At present, several techniques have been proposed for the encapsulation of probiotics, with the most common being extrusion, emulsification, and spray drying.

Extrusion is the oldest and most popular method for the encapsulation of probiotics. It involves extrusion of the cell/polymer suspension through a syringe needle or nozzle into a hardening solution, such as calcium chloride [6]. This method is appreciated for its simplicity, low cost, and gentle operations that retain a relatively high viability of probiotic cells [7]. The main disadvantage of this method is that it is unsuitable for large-scale production because of the slow formation of the capsules and the large size of the final microcapsules [8].

By contrast, spray drying is a relatively inexpensive, rapid, and suitable method for industrial scale-up [9]. In general, however, this method causes some injury to the encapsulated cells due to the use of dehydration and heating, which decrease the survivability of probiotic cells [10]. This method involves atomization of the cell/polymer suspension and its conversion into a spray of tiny drops that come into contact with a hot gas. The rapid evaporation of the solvent from the droplets causes their agglomeration into a dry powdered product [11].

Emulsification is another technique that is easily scaled up and produces small-sized beads (25 µm–2 mm). However, it is an expensive method because it requires some form of vegetable oil (soy, sunflower, corn, etc.) or paraffin oil for emulsion formation [4]. In this method, a small volume of cell/polymer suspension (as a dispersed phase) is added to a large volume of oil (as a continuous phase) [12].

All of these microencapsulation methods can also be used to incorporate a number of different nanomaterials that have recently gained interest because of their effects on the growth of agriculturally important plants. One example is carbon nanotubes (CNTs), which have demonstrated beneficial and stimulatory effects on plants and have enhanced crop yield [13]. Mondal et al. [14] studied the effect of CNTs on *Brassica juncea* at exposures of 2.3–46.0 μg/L and found enhanced germination, as well as increased root and shoot growth. Similarly, Wang et al. [15] reported 50% and 32% increases in the root length of wheat seedlings after three and seven days of exposure, respectively, to 40–160 μg/L CNTs.

The recent discovery of plant growth promoting rhizobacteria (PGPRs) now suggests new bacterial species that can be encapsulated to improve plant growth. These bacteria are presently being identified by next-generation sequencing (NGS), a new technique that can be applied to genomics studies and sequence analyses of individual genes, clusters of genes or operons, full chromosomes, or entire genomes of any organism [16]. Recently, NGS has been employed to study the genomes of several PGPRs, such as the probiotics *Pseudomonas* and *Bacillus* [17,18,19].

Whole genome analysis can provide a fundamental basis for future studies aimed at understanding the functions of organisms. Furthermore, comparisons among the completely sequenced probiotic bacteria genomes will help to offer new insights into evolutionary changes in these bacteria and highlight the genes that may contribute to plant growth promotion and biocontrol properties [20]. For example, Liu et al. [20] used whole genome analysis of *Klebsiella* sp. D5A, a plant growth stimulating bacterial species, to identify some genes related to plant growth-promotion responses, such as indole-3-acetic acid biosynthesis, phosphate solubilization, siderophore production, acetoin and 2,3-butanediol synthesis, and N_2_ fixation.

In the present study, we applied extrusion, emulsification, and spray drying techniques to entrap a *Pseudomonas* sp. strain (VUPF506) in an alginate–whey protein (ALG–WP) matrix. We compared the microcapsules produced by these methods to identify the best method for improving the survivability of strain VUPF506 and to assess the effects of ALG–WP–CNT microencapsulated strain VUPF506 in controlling *Rhizoctonia solani* infection in potato plants. Comprehensive genome-scale analyses resulted in species typing of *Pseudomonas* sp. VUPF506 as *Pseudomonas chlororaphis* and the identification of several secondary metabolite biosynthesis gene clusters in this strain.

## 2. Materials and Methods

### 2.1. Microorganisms

*Rhizoctonia solani* was obtained from the laboratory of Khorasan Razavi Agricultural and Natural Resources Research Center, and a *Pseudomonas* sp. strain (VUPF506) was selected from the biological control collection of the Vali-e-Asr University of Rafsanjan (this strain was chosen based on its known beneficial growth effects on potato plants).

### 2.2. Materials for Bacterial Microencapsulation

Sodium alginate (ALG) (M_w_ = 1.39 × 10^5^, β-d-mannuronate/α-l-guluronate ratio = 1.57) was purchased from Sigma-Aldrich (Taufkirchen, Germany). Whey protein concentrate (WPC80; 80% protein based on dry weight) was obtained from Alinda (Spata Attica, Greece). The carbon nanotubes (CNTs) were multi-walled carbon nanotubes with an outer diameter of 8–15 nm and a purity of >95 wt% (Neutrino Co., Tehran, Iran). Calcium chloride was obtained from Merck (Darmstadt, Germany), calcium carbonate from Iranian Nanomaterials Company (Mashhad, Iran), and soy oil from Sigma-Aldrich (Taufkirchen, Germany).

### 2.3. Encapsulation Process

Microencapsulation of the VUPF506 strain was carried out using three encapsulation techniques (extrusion, emulsification, and spray drying). For each microencapsulation technique, the number of cells was 2 × 10^11^ CFU/mL and the concentrations were obtained by optimization experiments.

#### 2.3.1. Extrusion Technique

An ALG–WP–CNT combination was prepared using the method of Rajam et al. [21] with some modifications. A 1.5% ALG solution containing 40 μg/mL CNTs was sterilized at 121 °C for 15 min and then added to an 8% WP solution prepared by dissolving 8 g WPC80 in 100 mL sterile distilled water. WP solution was stirred gently for 2 h, then let stand for 1 h, and solution was heated at 80 °C for 30 min. The bacterial solution was added to the polymer mixture, then beads were fabricated by extruding the bacterial cell-polymer solution into a 2% CaCl_2_ solution using a syringe with a diameter of 0.3 mm.

#### 2.3.2. Spray-Drying Technique

In this method, microcapsules were prepared by a spray-drying technique using WP, ALG, and CNT. A 1.5% ALG solution containing 40 μg/mL CNTs was mixed with 8% WP solution. The solution was stirred and mixed with the bacterial strain (the cell number was 2 × 10^11^ CFU/mL), followed by stirring in a homogenizer at 4000 rpm for 20 min. A laboratory-scale spray dryer (DorsaTech, Alborz, Iran) was used. At a steady flow rate of 4 L/h, the bacterial strain was atomized, causing vaporization of the surface moisture (about 2–5 s). Drying was performed at an inlet air temperature of 110°C and outlet air temperature of 60 °C. The vaporization rate was 3 L/h. Dried capsules were collected from the cyclone vessel, placed in a tightly sealed bottle, and stored in a desiccator.

#### 2.3.3. Emulsification Technique

The probiotics were microencapsulated by a modified emulsion method [22]. In brief, a 1.5% ALG solution containing 40 μg/mL CNTs was sterilized at 121 °C for 15 min and then added to an 8% WP solution. The solution was then mixed with 2 g calcium carbonate. After homogenization, the mixture was dispersed into twice the volume of soybean oil containing 2.5% (*w/v*) containing 0.2% Span 80 and emulsified for 15 min by stirring at 400 rpm. A 500 μL volume of glacial acetic acid was added, and stirring was continued for 15 min. The encapsulation process was completed by adding CaCl_2_. Microcapsules were collected by centrifugation at 300× *g* for 20 min and washed with distilled water.

### 2.4. Scanning Electron Microscopy of Microcapsules (SEM)

The surface morphology and particle size of the dry microcapsules were determined by Daypetronic Company using scanning electron microscopy (FESEM-Sigma VP, Zeiss, Germany). The average particle size was calculated from SEM images, and no less than 100 particles were used for the calculation.

### 2.5. X-ray Diffraction (XRD)

The XRD patterns for ALG, WP, and the ALG–WP–CNT microcapsules were recorded with an EQUNIOX300 diffractometer (INEL, Artenay, France) with a Cu Kα radiation source (λ = 1.54178 Å). Diffractograms were recorded from 10° to 80° (2θ) at an angular speed of 1°·min^−1^. The XRD patterns were graphed using Origin v 8.0 software (v 8.0, 2011, OriginLab, Northampton, MA, USA).

### 2.6. Determination of Encapsulation Efficiency

Microencapsulation efficiency was determined according to a modified method [23]. One gram of microcapsules was added into 10 mL phosphate buffer (pH = 7.4) and mixed for one hour. Then, the number of bacteria were counted by culture on nutrient agar medium. The number of entrapped bacteria (*N*) and the number of free viable bacteria cells before encapsulation in ALG–WP–CNT microcapsules (*N*_0_) were used in the following equation to estimate the encapsulation efficiency (*EE*):

*EE* (%) = *N*/*N*_0_ × 100

### 2.7. Release Behavior

In vitro release of viable cells of the encapsulated bacterial strain was determined using a dialysis bag method with some modifications [24]. In brief, 1 g of microcapsules was placed in a dialysis bag and immersed in phosphate buffer (pH = 7.4) for 60 days at room temperature (25 °C). The numbers of viable bacteria in the solution were enumerated by plate counting on nutrient agar.

### 2.8. Greenhouse Experiments

#### 2.8.1. Preparation of *R. solani* Inoculum

The pathogen was grown on potato dextrose agar for four days at 25 °C. Wheat grains (100 g) to be used as inoculum media were moistened with distilled water in a 500 mL flask and autoclaved at two-day intervals at 121 °C for 30 min. Five pieces (1 cm in diameter) of mycelial agar disk fungus cultures in dextrose agar (1 cm diameter) were inoculated onto sterile wheat grains and incubated for three weeks at 25 °C.

#### 2.8.2. Preparation of Antagonistic Suspensions

The VUPF506 bacterial strain was grown on nutrient agar medium and incubated for 24 h at 28 °C. The bacterial suspension was diluted in sterile, distilled water to a final concentration of 2 × 10^11^ CFU/mL, determined with a UV-Vis spectrophotometer U-2000 (Hitachi Instruments, Tokyo, Japan).

#### 2.8.3. Planting

The effect of the encapsulated and non-encapsulated VUPF506 bacterial strain against *R. solani* was tested on the Marfona potato cultivar, which is known to be sensitive to *R. solani*. Potato tubers were cleaned with tap water and sterilized with 0.5% sodium hypochlorite for 5 min. After rinsing in pure water twice, the tubers were sown in pots filled with a sand/soil mixture (pH 7.2, and EC 1.5 ds·m^−1^). All pots were maintained in a glasshouse at 22–25 °C for two months. The two-month-old potato plants were then inoculated with *R. solani* (5 g of pathogen inoculum per seedling) and microcapsules (1 g/kg soil).

#### 2.8.4. Disease Assessment

Six weeks after inoculation, the potato plants were washed free of soil, and visible lesions on their roots and crown were recorded. Lesions on the roots and crowns of the potato plants were evaluated, and disease severity was estimated using the following scale:

0 = no disease symptoms;
1 = small lesions (15 mm);
2 = large lesions (>15 mm);
3 = lesions girdling the stem.

The biocontrol efficacy was calculated by the following equation [25]:

Biocontrol efficacy (%) = ([disease incidence of control − disease incidence of treatment]/disease incidence of control) × 100

### 2.9. Genomic DNA Isolation

The VUPF506 strain was grown in tryptic soy broth medium at 28 °C for 24–48 h. Genomic DNA was extracted using a phenol–chloroform method. The extracted DNA was resolved on 0.8% agarose gel to check its integrity. The quality of the genomic DNA samples was assessed using a NanoDrop™ 2000 (Thermo Fisher Scientific Inc., Waltham, MA, USA).

### 2.10. Illumina Whole Genome Sequencing

This study used the HiSeq Illumina SBS technology platform (Illumina sequencing by synthesis technology; Macrogen, Inc., Seoul, Korea) to generate paired-end reads libraries for the VUPF506 strain. DNA libraries were obtained with the TruSeq Nano DNA Kit (Sample Preparation Guide, Part # 15041110 Rev. D). The quality of sequence reads was first analyzed using FastQC v0.11.9 (Available at: http://www.bioinformatics.babraham.ac.uk/projects/fastqc; accessed on 20 January 2021), and then the low quality reads (Phred quality score Q  <  30) were trimmed by Cutadapt v2.8 [26]. The VUPF506 strain was identified by comparing its similarity to three bacteria, confirmed as *P. fluorescens, P. putida,* and *P. chlororaphis*, following assembly of the sequencing reads with three genome references using BWA-mem v0.7.17.2. For better identification, genome assembly was carried out with the Unicycler v0.4.8.0 program and the obtained contigs were assessed with the Megablast program (Available at: http://www.ncbi.nlm.nih.gov/BLAST/; accessed on 30 January 2021). The obtained contigs were, finally, annotated using Prokka 1.14.6 and the antiSMASH program (Available at: https://antismash.secondarymetabolites.org; accessed on 28 February 2021) [27]. Data processing using Cutadapt, BWA-mem, and Unicycler software was conducted on the https://usegalaxy.eu/ web-site (accessed on 30 January 2021).

### 2.11. Phylogenetic Analysis

A phylogenetic tree was constructed based on the distances between the assembled contigs and the available strains using the Basic Local Alignment Search Tool (BLAST). The assembled contigs for *Pseudomonas* sp. VUPF506 were uploaded to the Type Strain Genome Server (TYGS) (available at: https://tygs.dsmz.de/, accessed on 28 February 2021) for taxonomic classification.

### 2.12. Statistical Analysis

Analysis of variance (ANOVA) was performed using the SAS program (SAS Institute, Cary, NC, USA). Significant differences of the means (*p* < 0.05) were determined by LSD’s Multiple Range Test.

## 3. Results and Discussion

### 3.1. SEM Morphology of the ALG–WP–CNT Microcapsules

**Figure 1:**
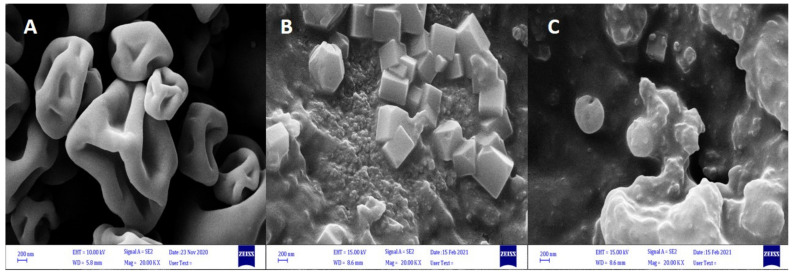
SEM micrographs of sodium alginate–whey protein–carbon nanotube (ALG–WP–CNT) microcapsules produced by (**A**): spray drying; (**B**): emulsification; (**C**): extrusion.

We obtained the ALG–WP–CNT microcapsules using three methods—extrusion (Section 2.3.1), spray drying (Section 2.3.2), and emulsification (Section 2.3.3). Figure 1 shows the shape and size of the microcapsules formed using different encapsulation techniques. Spray drying (Figure 1A) produced powder-like microcapsules with a diameter range of 1–10 μm, and most of those capsules had a spherical structure with concavities and wrinkles. Microcapsules produced by the emulsion method (Figure 1B) had non-homogenous shapes and morphology, with some showing cubic shapes (the cube crystals to be calcium carbonate), and others showing spherical shapes with smooth surfaces; their size range was 50–150 μm. The extrusion method produced spherical microcapsules with a size range of 150–250 μm. The microcapsules have a smooth surface with some encapsulated aggregates (Figure 1C). The reason for the observed concavities and wrinkles on the surfaces of the microcapsules was the high temperature of the walls of the drying chamber and the rapid water evaporation from the emulsion. These results are consistent with those reported by Banjare et al. [28].

Spherical microcapsules with smooth surfaces protect the main interior material and increase the microencapsulation efficiency. Distinct size differences were seen in the microcapsules produced by the different methods. Microcapsules derived from spray drying were typically small particles, while the extrusion method produced larger microcapsules. This is one of the main disadvantages of the extrusion method. Differences in size can arise due to the different parameters, such as nozzle diameter, stirrer speed, nozzle distance from the solution, gravity effect, solution composition and viscosity, and differences in the equipment used in the process of encapsulation, and these will all affect the microcapsule size [29].

### 3.2. XRD Structure of the ALG–WP–CNT Microcapsules

**Figure 2:**
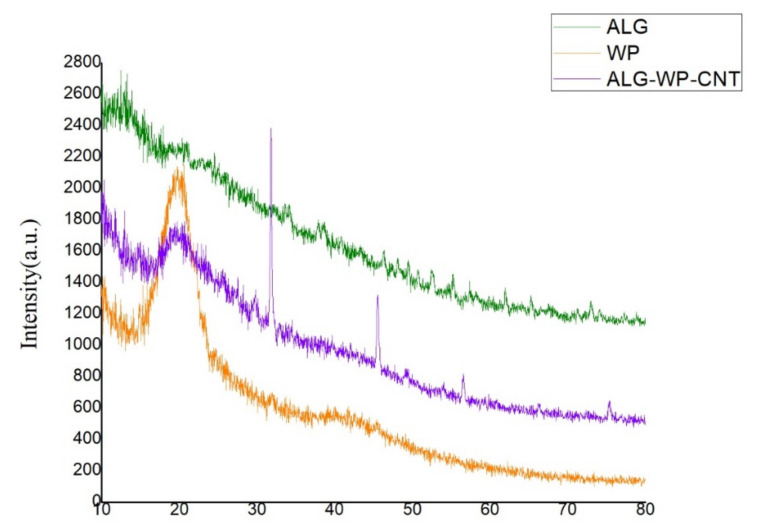
XRD patterns of sodium alginate (ALG), whey protein (WP), and ALG–WP–CNT microcapsules. Adapted from [5].

The XRD analysis was performed to study the amorphous and crystalline structure of the polymers used for microencapsulation. The XRD patterns for ALG, WP, and the produced microcapsules are shown in Figure 2. The ALG diffractogram did not show any crystalline peaks, whereas the WP diffractogram showed a reflection at 2 θ = 20°, demonstrating its crystalline nature [30]. The microcapsule XRD patterns revealed a decrease in peak height, confirming electrostatic interactions between WP amino acids and ALG carboxyl groups, in agreement with the results of Dehkordi et al. [30]. The microcapsule patterns showed a reflection at 2 θ = 26.44° associated with the CNTs; this was consistent with the results of Sasrimuang et al. [31]. Two reflections at 2 θ of approximately 32° and 45° could be explained by the presence of materials associated with the encapsulation process.

### 3.3. Encapsulation Efficiency

The use of CNTs in the microcapsule formulation was confirmed to be a promising strategy for increasing plant growth and development. Tests of the antibacterial effect of CNTs on the VUPF506 strain have shown no susceptibility of this strain to the antimicrobial effects in the presence of 40 µg/mL CNTs [5].

Results for the encapsulation efficiency confirmed that the viability of microencapsulated VUPF506 is clearly influenced by the encapsulation technique used (Table 1). The most important factor for probiotic encapsulation is the survival of the organism throughout the encapsulation process. The survival rate was higher for bacteria in microcapsules produced by the extrusion and emulsion techniques than for those for which the spray drying technique was used because of the milder conditions of the extrusion and emulsion microencapsulation methods. The comparatively low efficiency of encapsulation by spray drying was due to the increased inward airflow temperature, which causes thermal inactivation and dehydration of the cells. High temperatures can damage cellular components, such as DNA, RNA, proteins, and ribosomes [32].

### 3.4. Release Patterns

**Figure 3:**
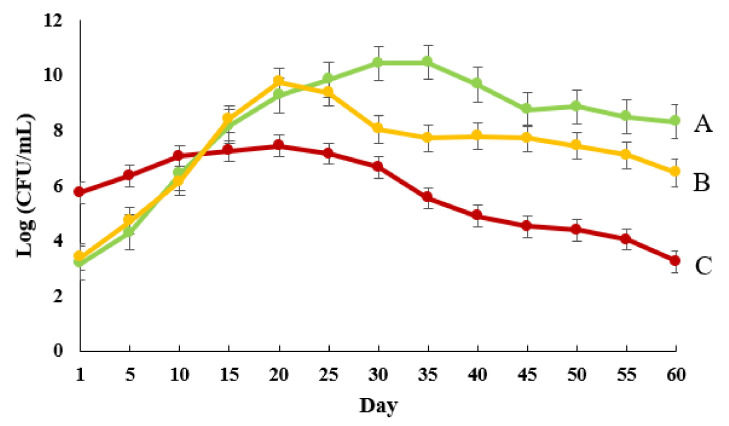
Release behavior of *Pseudomonas chlororaphis* VUPF506 encapsulated in ALG–WP–CNT microcapsules using different techniques. Error bars represent standard error *(n* = 3). (**A**: Extrusion; **B**: emulsion; **C**: spray drying).

Figure 3 shows the bacterial release rate from ALG–WP–CNT microcapsules produced by the three different methods. The microcapsules produced by spray drying showed a more rapid release in the initial days compared with the other two methods. The burst release from the spray-dried microcapsules could reflect the release of bacteria present on the surface of the microcapsules, as these would quickly enter the release environment. Notably, the lower bacterial population in these microcapsules could indicate the death of a large number of bacteria during the spray-drying process. By contrast, the release rate was gradual for the extrusion and emulsion-based microcapsules. The maximum release was recorded on the 20th and 35th day for the emulsion and extrusion methods, respectively. Following these time points, the release rate was lower and showed a stable trend until the end of the experiment. Gradual release leads to better bacterial establishment and root colonization. Van Elsas et al. [33] found that encapsulation of *Pseudomonas fluorescens* in ALG beads improved the inoculant effectiveness and colonization of wheat roots.

### 3.5. Greenhouse Results

**Figure 4:**
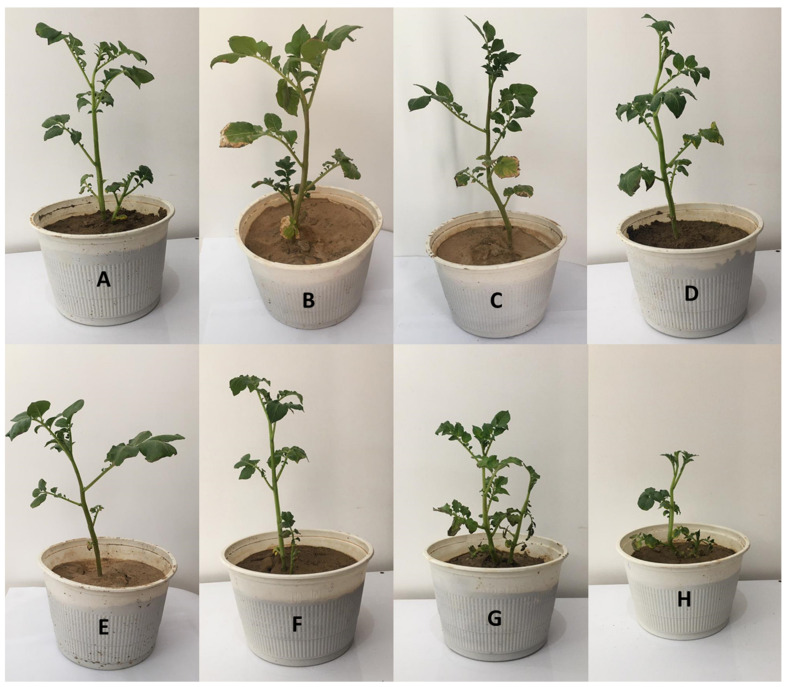
The effect of bacterial strain *Pseudomonas chlororaphis* VUPF506 and its ALG–WP–CNT microcapsules on the control of *Rhizoctonia solani* in potato plants. (**A**): Spray drying-based microcapsules + pathogen, (**B**): extrusion-based microcapsules + pathogen, (**C**): emulsification-based microcapsules + pathogen, (**D**): microcapsules without bacterial strain + pathogen, (**E**): *P. chlororaphis* VUPF506, (**F**): *P. chlororaphis* VUPF506 + pathogen, (**G**): control, (**H**): pathogen.

**Figure 5:**
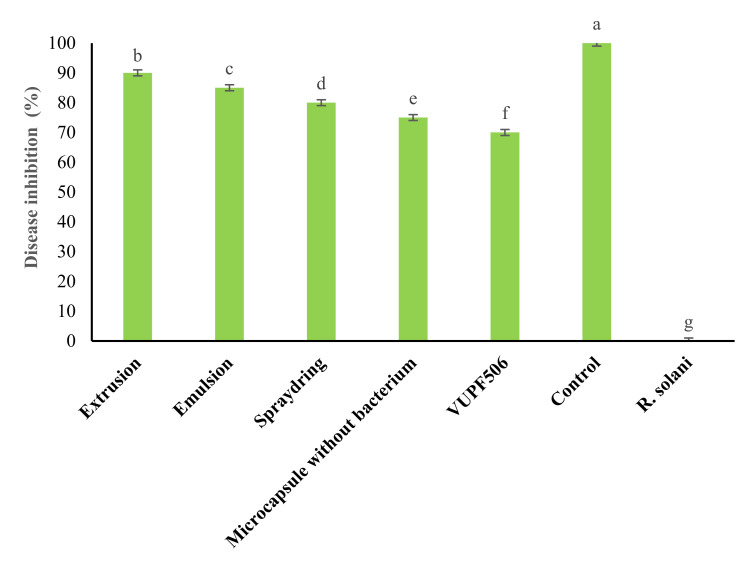
Effect of treatment of potato plants with ALG–WP–CNT microcapsules produced by extrusion, emulsification, and spray drying, and containing *Pseudomonas chlororaphis* VUPF506, on disease inhibition under greenhouse conditions. The experiment was arranged as a factorial in the framework of a completely randomized design with three replications. Error bars represent standard error, and letters indicate a statistical difference compared to the control (*p* < 0.05). Means with the similar letters in the same columns are not significantly different at 5% of probability level based on Duncan Multiple Rang Test.

**Figure 6:**
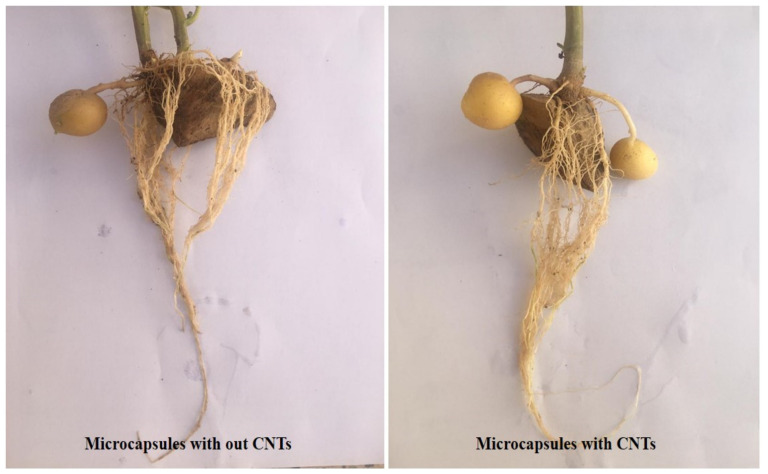
Effect of encapsulation of carbon nanotubes into ALG–WP microcapsules on potato plants. The extrusion method was used for preparation of microcapsules. **Left panel**: plant treated with ALG–WP microcapsules. **Right panel**: plant treated with ALG–WP–CNT microcapsules.

As shown in Figure 4, all treatments reduced *R. solani* disease intensity in potato plants. Among the microencapsulation treatments, the extrusion method gave the highest rates of disease control, at 90% (Figure 5). Generally, in addition to disease intensity reduction, the treatment of potato plants with microcapsules produced by the different methods enhanced plant growth parameters, such as plant height, leaf number, leaf area size, and vegetation index. These effects can be attributed to the WP, as it has many amino acids that can contribute greatly to plant physiological activities. Different types of amino acids can also induce resistance in plants by strengthening their defense systems against plant pathogens [5]. The VUPF506 bacterial strain is known to reduce disease severity due to its high biocontrol capacities, including extracellular enzyme secretion, plant growth hormone production, and resistance induction in plants. The use of CNTs in the microcapsule coating increased the growth of roots and encouraged the production of potato tubers (Figure 6). Cañas et al. [34] showed that the application of CNTs increased the root length in onion and cucumber. Moradi-Pour et al. [35] showed that CNTs increased root mass and potato tuber production. They concluded that the presence of CNTs in nanoencapsulations leads to stronger root systems and improved absorption of water and nutrients.

### 3.6. Illumina Genome Sequence

The main purpose of the whole genome sequencing of the VUPF506 strain was to accurately identify the strain. The whole genome of strain VUPF506 contained 22,065,448 raw reads, with average read lengths of 151 bp. The average GC content was 63%. When reference genomes are available, an assembly can be made by comparison or direct mapping. Based on the very similar biochemical test results, the complete genome sequences of three bacterial species (*P. fluorescens, P. putida,* and *P. chlororaphis*) were downloaded from GenBank. The mapping percentage sequencing was significantly higher for *P. chlororaphis* (96.5%) than for *P. fluorescens* (46.3%) or *P. putida* (33.59%) (Table 2), indicating a close similarity of the VUPF506 genome to the genome of *P. chlororaphis*. De novo assembly, which is used to reconstruct the genome without prior information of the organism, was also carried out by assembling the sequencing reads without the genome reference using a de novo assembler (Unicycler). The length of the largest contig created by Unicycler was 486,020 bp, with an N50 value of 153,721 bp (Table 3). The longest contig showed the highest similarity with the species *P.* *chlororaphis* (CP008696.1) following the creation of an alignment with a length of 261,201 bp at a rate of 99%. All the tested methods confirmed this strain as *P. chlororaphis*.

### 3.7. Phylogenetic Analyses

**Figure 7:**
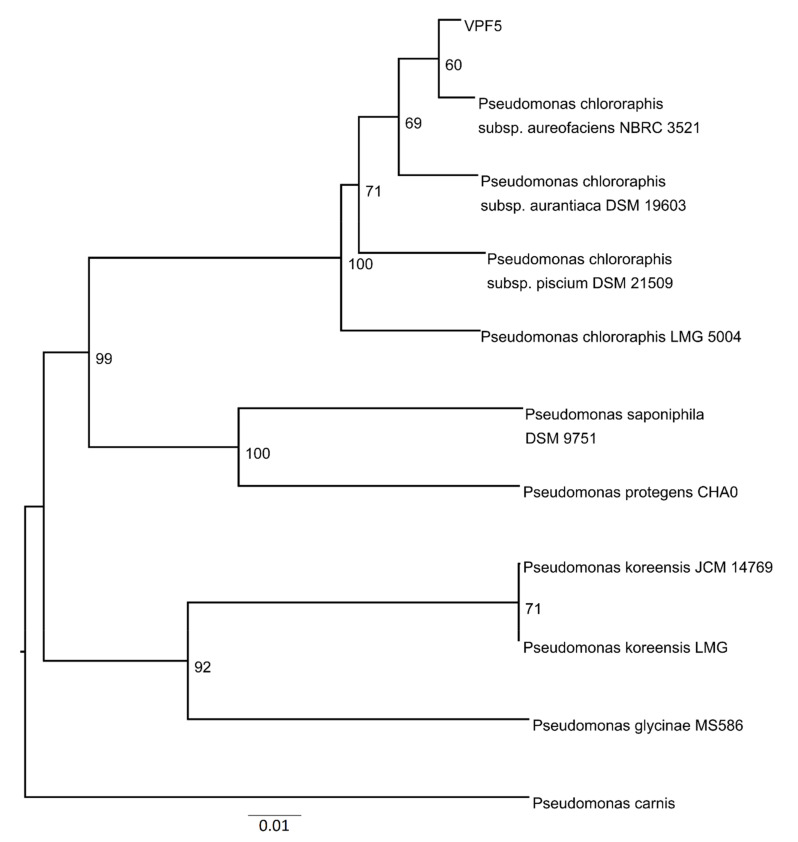
The distance-based phylogenomic tree based on the genomes of VPF506 and 10 *Pseudomonas* type strains retrieved from TYGS server (available at https://tygs.dsmz.de/, accessed on 20 January 2021). The tree was inferred using FastME2.1.6 [36] by calculating Genome BLAST Distance Phylogeny (GBDP) among selected genome sequences. The pseudo-bootstrap support value from 100 replicates is indicated per each internal node and the branch lengths are scaled using GBDP distance formula d5.

Figure 7 shows the phylogenetic tree for VUPF506. Here, the 20 contigs with the largest lengths were selected and type strains of relevance were investigated by the TYGS based on the genetic distance and with the BLAST program. TYGS compared the VUPF506 strain genome against its database of type (strain) genomes by incorporating the techniques of the Genome-to-Genome Distance Calculator. The TYGS analysis of the *Pseudomonas* sp. VUPF506 contigs revealed that this strain belongs phylogenetically to *P. chlororaphis.*

## 4. Conclusions

Microencapsulation is a promising technology for protecting the potency of probiotic bacteria and facilitating their controlled release into the soil. According to our study, ALG–WP–CNT microcapsules improve the stability and efficiency of probiotic bacteria and lead to their gradual and controlled release. The present study examined the effect of common encapsulation methods (extrusion, emulsification, and spray drying) on bacterial survival. The extrusion method was particularly effective at increasing the efficacy and viability of probiotic bacteria because it provides the mildest conditions during the process of microencapsulation. The application of this novel ALG–WP–CNT formulation in agriculture should lead to the generation of biofertilizers that improve the viability of probiotic bacteria and retain their biocontrol properties. These encapsulated probiotics can then be utilized to enhance plant growth and reduce the negative effects of pathogens on plants.

## Figures and Tables

**Table 1:** Encapsulation efficiency of VUPF506 into ALG–WP–CNT microcapsules.

Encapsulation Technique	Encapsulation Efficiency (%) *
Extrusion	86.1 ± 1.2 ^a^
Emulsification	81.7 ± 0.5 ^b^
Spray drying	77.5 ± 1.0 ^c^

* Mean ± standard error. ^a–c^ Significant differences are according to Student’s *t*-test with *p* < 0.05.

**Table 2:** The results of mapping to reference genomes.

	*P. fluorescens*	*P. putida*	*P. chlororaphis*
Number of mapped sequences	21,062,282	7,294,692	10,071,541
Mapped sequences (%)	96.5	33.59	46.3

**Table 3:** Genome assembly statistics.

	Create Assemblies with Unicycler
# contigs (≥0 bp)	78
# contigs (≥1000 bp)	63
Total length (≥0 bp)	6,483,708
Total length (≥100 bp)	6,480,725
# contigs	63
Largest contig	486,020
Total length	6,480,725
GC%	6332
N50	153,751
N75	106,268
L50	12
L75	24
#N’s per100 kbp	0.00

## Data Availability

Not applicable.
